# The effects of cynaroside on lipid metabolism and lipid-related diseases: a mechanistic overview

**DOI:** 10.3389/fphar.2025.1648614

**Published:** 2025-07-31

**Authors:** Chenyi Cen, Jiahuan Li, Pu Zhou, David Fisher, Nguyen Thi Thu Hien, Erkin Musabaev, Khrystyna Pronyuk, Lei Zhao

**Affiliations:** ^1^ Department of Infectious Diseases, Union Hospital, Tongji Medical College, Huazhong University of Science and Technology, Wuhan, China; ^2^ Department of Medical Biosciences, Faculty of Natural Sciences, University of the Western Cape, Cape Town, South Africa; ^3^ Hai Phong University of Medicine and Pharmacy, Hai Phong, Vietnam; ^4^ The Research Institute of Virology, Ministry of Health, Tashkent, Uzbekistan; ^5^ Infectious Diseases Department, O.Bogomolets National Medical University, Kyiv, Ukraine

**Keywords:** cynaroside, lipid metabolism, lipid-related diseases, pharmacological effect, clinical application

## Abstract

Cynaroside is a natural flavonoid compound, which is widely found in plants. It has the effects of lowering fat, anti-diabetes, anti-inflammatory, antioxidant, anticancer, antibacterial and liver protection. Recent studies have shown that cynaroside regulates fat metabolism through multiple mechanisms, including modulating lipase activity, enhancing gut health and suppressing inflammatory responses. These processes involve the NF-κB, NLRP3 and JAK/STAT inflammatory pathways, and other signaling pathways. By controlling complications associated with abnormal fat metabolism, cynaroside has been demonstrated therapeutic effects on obesity, fatty liver disease, type 2 diabetes and other conditions. Therefore, it shows great potential as an alternative treatment for lipid metabolism-related diseases. However, although the extraction method of cynaroside has been mature, the study of its monomer is still in the initial stage, and there is no complete human efficacy and safety evaluation report. This paper introduces the molecular structure, source and pharmacological action of cynaroside, and systematically reviews the mechanism of regulating lipid metabolism of cynaroside, so as to expand the application value of cynaroside. In addition, it also puts forward the challenges, solutions and future research directions in the clinical application of cynaroside.

## 1 Introduction

Cynaroside, also known as luteolin-7-O-glucoside, is a natural flavonoids compound. It was originally isolated from the plant (*Reseda odorata* L.) (Ke et al., 2011) of the family (Resedaceae) and has subsequently been widely found in various plant taxa, including the genera *Lonicera* (e.g., *Lonicera japonica* Thunb.), *Chrysanthemum*, and *Taraxacum*, as well as members of the families Apiaceae, Poaceae, Lamiaceae, Solanaceae, Zingiberaceae, and Asteraceae (Jiang et al., 2010). Cynaroside has been reported as one of the main chemical constituents of *Cynara scolymus* L., which is also known as artichoke and extensively cultivated in Mediterranean region, African and American countries (Emendörfer et al., 2005). The sprout of *C. scolymus* is commonly used as a vegetable, and its leaves exhibit a broad spectrum of biological activities and have significant potential for applications in nutrition and health products, agricultural chemicals, pigments and food additives (Nassar et al., 2013). In honeysuckle, the content of cynaroside is relatively high, reaching up to 0.09% (Ke et al., 2011). Cynaroside has been reported to exert anti-inflammatory and antioxidant activities, as well as inducing apoptosis of different types of cancer cell, and can be used for the treatment of dyspeptic, hepatitis, hyperlipidemia, obesity disorders and many other diseases (Ma et al., 2024). Recently, beneficial effects of cynaroside in the immune system and chronic metabolic diseases have been also widely reported (Orhan et al., 2016; Vo Van et al., 2022).

Lipid metabolism refers to the process of digestion, absorption, synthesis, and decomposition of fats in living organisms by various enzymes. The metabolites include adipokines, fatty acids, and cholesterol substances (Jeon and Carr, 2020). Lipids are not only the basic component of biological membrane structure, but also dynamically regulate the metabolic homeostasis by participating in signal transduction, β-oxidation, energy storage and energy supply (Terry and Hay, 2024). Under physiological conditions, lipid metabolism plays an important role in human growth and development, metabolism, and tissue reconstruction (Tvrzicka et al., 2011). However, long-term exposure to high-fat diet (HFD), environmental toxicants (such as tobacco cadmium) or chronic disease states can lead to an imbalance between lipid intake and consumption, induce molecular events such as endoplasmic reticulum stress, mitochondrial dysfunction and oxidative stress, and eventually form a “lipotoxic” effect, causing lipid-related diseases (Dong et al., 2024). Lipid-related diseases refer a variety of chronic diseases of circulatory system and tissue lipid accumulation, including hyperlipidemia, obesity, type 2 diabetes (T2DM), metabolic dysfunction-associated fatty liver disease (MASLD), and atherosclerotic cardiovascular disease, which are usually accompanied by reduced plasma high-density lipoprotein cholesterol (HDL-C) levels, and elevated triglycerides (TG), total cholesterol (TC) and low density lipoprotein cholesterol (LDL-C) concentration (Katsiki et al., 2016). According to the latest forecast of the World Obesity Federation, to 2035, global overweight/obese population will exceed 51%, and the corresponding total number of individuals will be more than 4 billion. Obesity among adolescents is on a significant upward trend, with the prevalence of obesity among males under 18 years of age expected to double to 208 million, and the number of girls in the same age group will grow even faster, by 125 percent, resulting in 175 million girls suffering from obesity (World Obesity Federation, 2025). Thus, lipid homeostasis imbalance and lipid-related diseases have become non-negligible challenges threatening global public health.

Current clinical interventions of lipid metabolism and related metabolic disorders mainly rely on chemical synthesis drugs. Statins reduce cholesterol synthesis by inhibiting 3-Hydroxy-3-methylglutaryl coenzyme A reductase (HMG-CoAR), but may cause liver enzyme abnormalities and myopathy (Sirtori, 2014). Omega-3 polyunsaturated fatty acids can regulate TG metabolism, but they are costly and have individual difference in efficacy (Shahidi and Ambigaipalan, 2018). The limitations of these therapies drive the exploration of novel regulatory strategies, such as microecological modulators targeting gut microbiota-host interactions, and precision nutritional interventions based on lipidomics (Schoeler and Caesar, 2019). It is worth noting that the molecular mechanism analysis of lipotoxicity-related signaling pathways, e.g., sterol regulatory element binding protein (SREBP), peroxisome proliferator-activated receptor (PPAR) provides a theoretical basis for the development of targeted drugs with low side effects (Zhang et al., 2018).

Cynaroside is one of the most common natural and safe bioactive substances and has attracted wide attention for its various biological functions such as anti-oxidation, antibacterial, anti-inflammatory, antiviral, anti-cancer and regulation of lipid metabolism (Bouyahya et al., 2023). Amounts of studies have shown that cynaroside plays a critical role in preventing and regulating lipid metabolism disorders (Azevedo et al., 2010; Orhan et al., 2016). With the increasing incidence of chronic diseases and rising medical costs, the value of cynaroside is prominent. However, recent reports of adverse effects of traditional Chinese medicine are also increasing, and toxicological research on cynaroside is in its infancy. Therefore, this paper aims to explore pharmacology, toxicity, pharmacokinetics, and the mechanism of cynaroside in regulating lipid metabolic processes and its prospects for clinical application in lipid-related diseases, with a view to providing valuable insights into its therapeutic potential.

## 2 The structure, molecular formula, source of cynaroside

**FIGURE 1:**
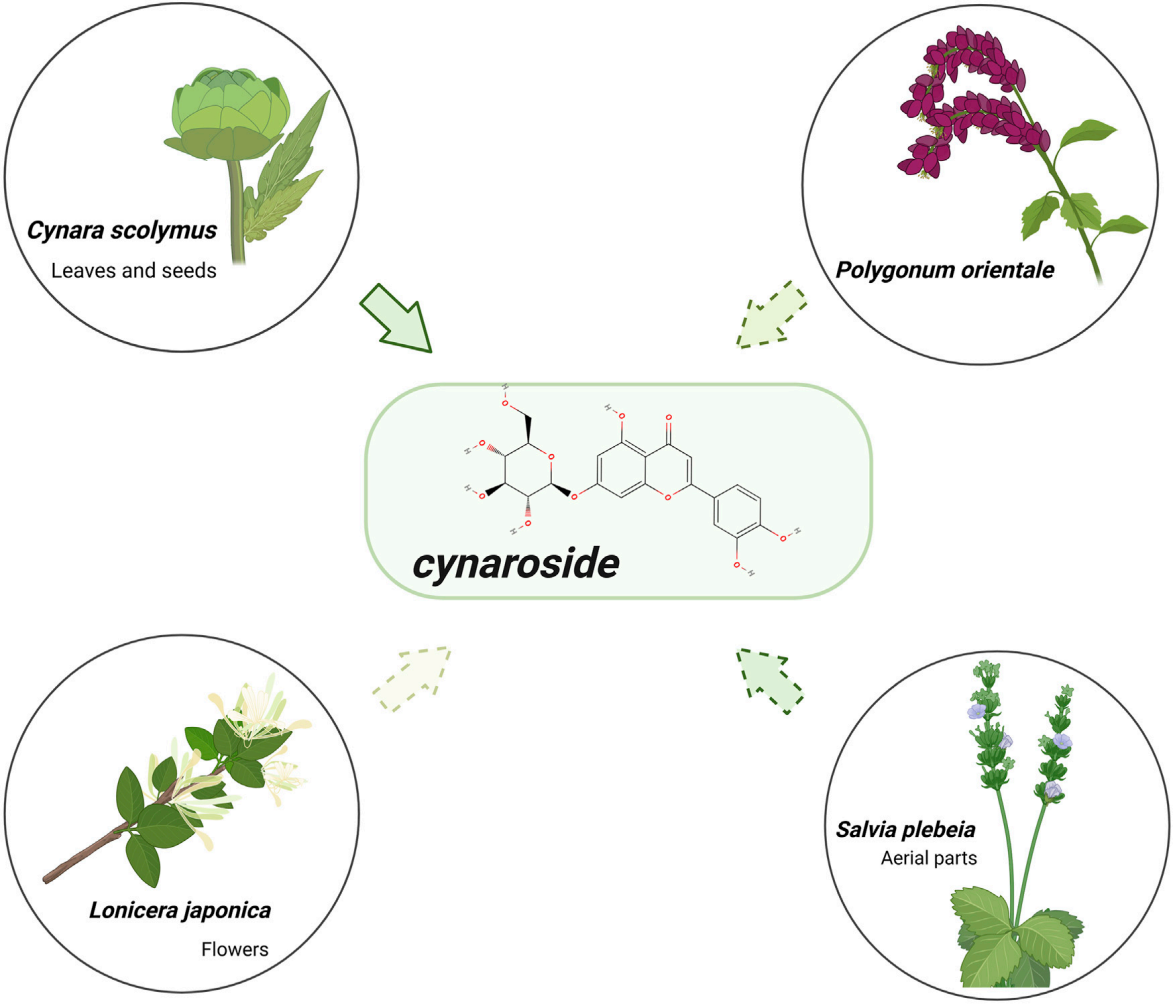
Chemical structure and natural source of cynaroside. Cynaroside was harvested in natural sources, mainly from the leaves and seeds of Cynara scolymus and flowers of *Lonicera japonica*, followed by the aerial parts of Salvia plebeia and Polygonum orientale.

Cynaroside (PubChem CID: 5280637, CAS number: 26811-41-6, MW: 448.4 g/mol,), with the molecular formula C21H20O11, is a flavonoid compound widely found in plants and has various names (luteolin-7-O-glucoside, luteoloside, cinaroside) (Information, 2024). The chemical structure of cynaroside is shown in Figure 1. It is abundant from natural sources and can be extracted from seeds, roots, stems, leaves, bark, flowers, fruits, aerial parts, and whole plants of several umbiaceae, Poaceae, Lamiaceae, Solanaceae, Asteraceae and other families (Bouyahya et al., 2023) (Figure 1).

Cynaroside is not only a major component of *C*. *scolymus*, but also in many other extracts from parts of plants, most of which are grown in Southeast Asia. In traditional Chinese medicine, cynaroside is the indicator component of honeysuckle quality control in Chinese Pharmacopoeia and the main component of honeysuckle exerting pharmacological action (Ke et al., 2011). In China, cynaroside is mainly harvested from *Cuminum cyminum* L. (Zhang et al., 2011), the extract of *Prunus pseudocerasus* Lindl. (Dong et al., 2021), *L*. *japonica* flos (Liu et al., 2019), *Elsholtiza bodinieri* Vaniot (Zou et al., 2018) and *Polygonum orientale* L. (Huang et al., 2013). It has also been found in many neighbouring Asian countries. Cynaroside can be collected from the extracts like *Anthriscus sylvestris* (L.) Hoffm. (Hong et al., 2021), *Chrysanthemum morifolium* Ramat. (Suh et al., 2020), *Salvia plebeia* R. Br. (Lee et al., 2018), *Lonicera japonia* (Nho et al., 2018), *S*. *plebeia* leaves (Nugroho et al., 2012), *Ixeris dentata* (Thunb.) Nakai roots (Lee et al., 2008) and *Angelica keiskei* (Miq.) Koidz. from Korea (Park et al., 2002). And cynaroside has been isolated from *Sonneratia caseolaris* (L.) Engl. part extracts in Japan (Sadhu et al., 2006).

## 3 The extraction process of cynaroside

Through summarizing recent research reports, six extraction methods are summarized, including three traditional extraction methods: solvent extraction, ultrasonic extraction and flash extraction (Ke et al., 2011). And three emerging extraction methods: ultra-high pressure extraction, supercritical fluid extraction and ultrasonic assisted extraction. 1. Solvent extraction: The efficient extraction of cynaroside was mainly carried out by solvent extraction, among which ethanol water solution extraction technology was particularly prominent (Lama-Muñoz et al., 2019). Because its method is simple, equipment cost is low and suitable for large-scale production, it is still the mainstream of industrial production. 2. Ultrasonic extraction: The reflux extraction method in solvent extraction is generally carried out at a high temperature, which is easy to affect the activity of effective components or lead to the loss of effective components (Lijun et al., 2017). Men et al. optimized the effects of two extraction methods, such as comparative heating reflux and ultrasonication, on chlorogenic acid and cynaroside in honeysuckle medicinal materials. The results showed that ultrasonication was slightly better than reflux method with cynaroside transfer rate as the index (Chunyan et al., 2011). 3. Flash extraction: Compared with the first two extraction methods, flash extraction can extract bioactive components quickly and efficiently. However, the defects of this method are also very obvious. It is only suitable for soft raw materials (such as plants, flowers and leaves), and woody roots and stems need pre-pulverization. Moreover, the preparation cost is high and the processing capacity is limited (Lijun et al., 2017).

The three emerging extraction technologies all have the disadvantages of expensive equipment and complex operation. 1. Ultra-high pressure extraction: Researchers employed both conventional dynamic maceration and a Ultra-high pressure extraction technique for olive leaf extraction. Through central composite experiments and Box-Behnken experimental design, they systematically optimized key parameters including temperature, leaf moisture content, solvent/gross weight ratio, and ethanol/water solution concentration. The results demonstrated that the Ultra-high pressure extraction method significantly outperformed traditional dynamic maceration in extracting olive bitter oleuropein and cynaroside (Lama-Muñoz et al., 2019). 2. Upercritical fluid extraction: Commonly used CO2 as a supercritical fluid. The advantages are environmental protection, non-toxic, good selectivity, no solvent residue in the product, and is often used to make high purity drugs. Villalva et al. suggest that supercritical fluid extraction using pure carbon dioxide is considered a green technology for obtaining plant extracts with potential antioxidant and anti-inflammatory activities. Supercritical fluid extraction was demonstrated higher total phenolic content and antioxidant activity levels (Villalva et al., 2021). 3. Ultrasonic assisted extraction: Hao et al. optimized the conditions of ultrasonically assisted extraction of flavonoids. It was concluded that ultrasonic assisted extraction had the strongest extraction capacity and the highest efficiency compared with solvent extraction and microwave extraction in terms of the yield of flavonoids (Hao et al., 2023).

## 4 Pharmacology of cynaroside in lipid metabolism and related diseases

**FIGURE 2:**
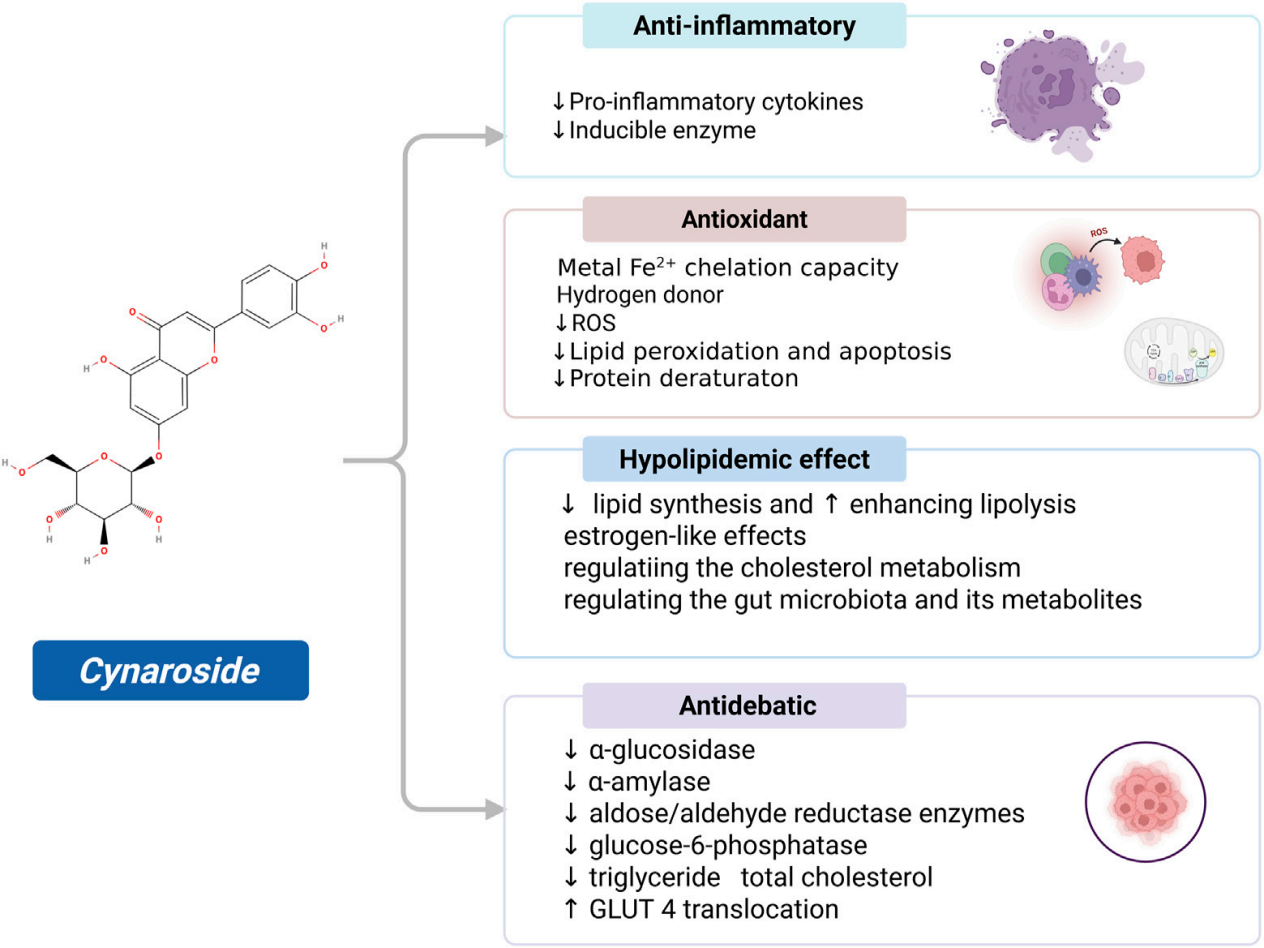
Pharmacology of cynaroside in lipid metabolism and related diseases. Cynaroside ameliorates lipid metabolism disorders through synergistic mechanisms: Its anti-inflammatory properties inhibit the production of pro-inflammatory cytokines and inducers, while exerting potent antioxidant effects against oxidative stress via its self-chelating hydrogen donors and ferrous ions. The compound regulates glucose metabolism-related enzymes to improve glucose homeostasis, and inhibits abnormal lipid accumulation by suppressing fat synthesis and promoting fat breakdown, thereby providing comprehensive protection against metabolic diseases.

The pharmacological effects of cynaroside in lipid metabolism and related diseases include antioxidant, anti-inflammatory, anti-debatic and anti-lipemics properties (Figure 2). It has also been the subject of numerous investigations due to its beneficial health properties, which justify its frequent study.

### 4.1 Hypolipidemic effect of cynaroside

**FIGURE 3:**
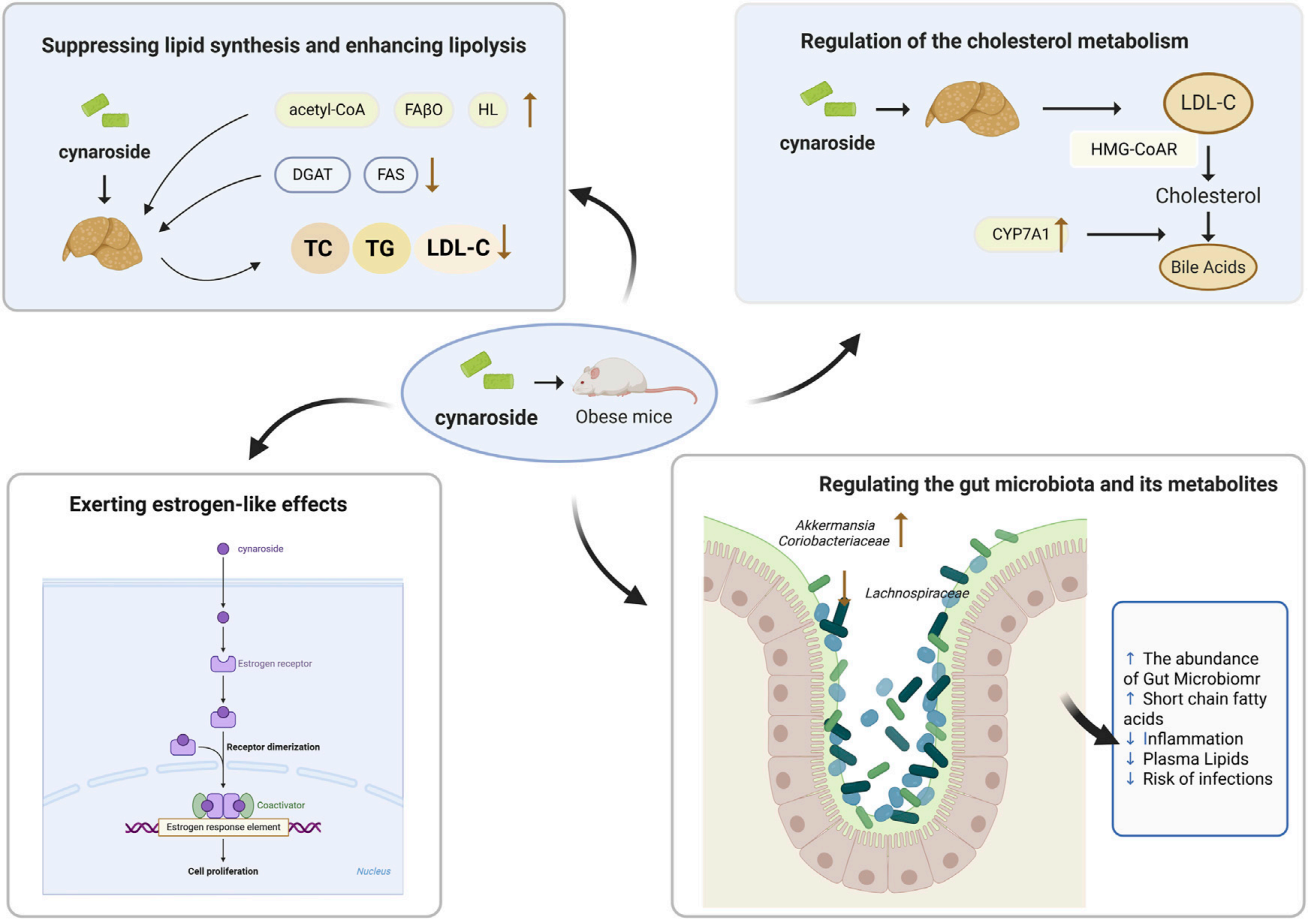
Hypolipidemic effect of cynaroside. The four major action points of Cynaroside in lowering lipid are: (1) regulation of cholesterol metabolism; (2) inhibition of lipid synthesis and promotion of fat breakdown; (3) estrogen-like effect; (4) regulation of intestinal flora and metabolites (Abbreviation: ACACA, Acetyl-CoA carboxylase Acetyl CoA carboxylase; FAβO, Fatty acid β-oxidase; HL, Hepatic Lipase; DGAT, Diacylglycerol acyltransferase; FAS, Fatty Acid Synthase; TC, Total Cholesterol; TG, Triglycerides; LDL-C, Low-Density Lipoprotein Cholesterol; CYP7A1, Cholesterol 7α-Hy-droxylase).

Hypolipidemic effect of cynaroside involves four aspects: regulation of the cholesterol metabolism, suppressing lipid synthesis and enhancing lipolysis, reducing blood lipid by exerting estrogen-like effects, and regulating the gut microbiota and its metabolites (Figure 3).

#### 4.1.1 Regulation of the cholesterol metabolism

Cholesterol metabolism includes the process of cholesterol synthesis, esterification, uptake, intracellular transport and excretion. Hepatocytes are important cells involved in liver cholesterol metabolism, which regulate lipid metabolism by regulating the activity of a variety of enzymes in the body and maintaining the stability of intestinal microorganisms and their metabolites (Musso et al., 2013). The primary sources of hepatic cholesterol are derived from the uptake of plasma lipoproteins and *de novo* synthesis processes. HMG-CoAR is regulated by transcription factors such as SREBP-2, which is the rate-limiting enzyme in cholesterol biosynthesis (Zhang et al., 2016). When consuming high-fat foods, SREBP-2 is activated and binded to sterol regulatory elements, thereby affecting the expression level of HMG-CoAR (Langston et al., 2005). Gebhardt (1998) firstly found that cynaroside extracting from artichoke leaves inhibited the cholesterol biosynthesis of 14C-acetate in a time-dependent manner in primary cultured rat hepatocytes. In addition, cynaroside was indirectly reversible and time-dependently suppressed HMG-CoA, thus effectively blocking the effects of insulin on cholesterol biosynthesis (Gebhardt, 1998). This finding was later confirmed through animal experiments by Azevedo et al. (2010).

Cholesterol 7α-hydroxylase (CYP7A1) is a rate-limiting enzyme that transforms cholesterol in non-hepatic peripheral tissues into bile acids (BAs) by catalyzing the synthesis of hepatic BAs (Ge et al., 2019). Sun et al. (2021) found that the enzyme activities related to lipid synthesis of HMG-CoAR were significantly decreased and CYP7A1 activities were significantly increased after the intervention of cynaroside, implicating the potential of cynaroside in the regulation of cholesterol metabolism.

#### 4.1.2 Suppressing lipid synthesis and enhancing lipolysis

Lipid metabolism is a complex process that is regulated by various organs, such as the liver and small intestine (DeBose-Boyd, 2018). Fat synthesis and catabolism in the liver involve many key enzymes. Fatty acid synthase (FAS) plays a key role in lipogenesis as a central synthase of lipid metabolism (Fhu and Ali, 2020). Fatty acid β-oxidase (FAβO) is a key enzyme in fatty acid catabolism, and promotes the β-oxidation of fatty acids to regulate lipid metabolism (Cherkaoui-Malki et al., 2012). Increased activation of fatty acids and reduced β-oxidation are important contributors to lipid deposition in the liver (Li et al., 2012). Hepatic lipase (HL) is a congenital liver enzyme that promotes TG clearance from very low-density lipoprotein (VLDL) pools, but HL release and transport are controlled by HDL (Chatterjee and Sparks, 2011). Diacylglycerol acyltransferase (DGAT) is the last rate-limiting enzyme for the synthesis of triacylglycerol and mainly catalyzes the binding of diglycerides to fatty acyl groups (Bhatt-Wessel et al., 2018). Sun et al. (2021) established a HFD-induced rat model to study the mechanism of hypolipidemic effect of cynaroside and found significant decreases in body weight, TC, TG, and LDL-C in cynaroside-treated rats compared to the control group. Enzyme-linked immunosorbent assay (ELISA) analysis found that the enzyme activities related to lipid synthesis of FAS and DGAT were significantly decreased, while the FAβO and liver lipase activities were significantly increased. Xiao et al. (2022) further found that the hypolipidemic mechanism of cynaroside mainly involves fatty acid metabolism with the significant downregulation of Lpin 1 and remarkable upregulation of Acetyl-CoA carboxylase Acetyl CoA carboxylase (ACACA). ACACA is a key gene for *de novo* fatty acid synthesis, and down-regulating its expression can deeply inhibit the biosynthesis of fatty acids (Dankel et al., 2021). The study on 3T3-L1 cells and HepG2 hepatocytes has shown that phosphatidic acid phosphohydrolase1 (LPIN1) plays a key role in adipogenesis, acting as a co-activator of peroxisome proliferator-activated receptor gamma coactivator 1a (PGC-1a) to regulate fatty acid metabolism (Zhou et al., 2022).

#### 4.1.3 Reducing blood lipid by exerting estrogen-like effects

Phytoestrogens are a class of plant substances or metabolites that can mimic or modulate the action of endogenous oestrogens, including non-steroidal phenolic compounds and steroidal phytosteroids (Mazur and Adlercreutz, 1998). They induce or regulate estrogen signaling pathways by binding to estrogen receptors, including kinase activation and transcription gene regulation. The chemical structure of phytoestrogens has a remarkable characteristic structure, the phenolic ring, which is a prerequisite for binding to the oestrogen receptor (Leclercq and Heuson, 1979). Estrogen plays a key protective role in the development of obesity and metabolic diseases by regulating various metabolic processes, including glucose and lipid metabolism, body weight, adipose tissue distribution, caloric intake, and energy expenditure (Ribas et al., 2010). Ammar et al. found that cynaroside had a significant estrogenic effect, maintained normal uterine weight, increased plasma estradiol levels, suppressed bone turnover markers, and improved plasma lipid profile. In animal experiments, cynaroside was comparable to estradiol in improving plasma lipid profile in ovariectomized rats (Ammar et al., 2016).

#### 4.1.4 Regulating the gut microbiota and its metabolites

The intestinal flora composed of thousands of bacteria is closely related to the health of human body. Several studies have reported that many metabolic diseases, including obesity, diabetes and hyperlipidemia, are associated with intestinal dysbiosis (Lim et al., 2016; Wang et al., 2021). The gut bacteria are predominantly composed of *Firmicutes*, *Bacteroidetes*, *Proteobacteria* and Actinobacteria phyla, with *Enterobacteriaceae*, *Bacillaceae*, *Bacteroideaceae* and *Actinomycetaceae* being the dominant classes, comprising approximately 90% (Le Chatelier et al., 2013). It has been shown that a HFD significantly changed the composition of the intestinal flora, increased the relative abundance of *Firmicutes* and decreased the relative abundance of *Bacteroidetes*, resulting in the increase of *Firmicutes* to *Bacteroidetes* ratio (F/B ratio) (Daniel et al., 2014).

Short-chain fatty acids (SCFAs) are the most common metabolite produced by the intestinal flora and derived from the anaerobic digestion of undigested carbohydrates in the colon, including acetic acid, propionic acid, butyric acid, isobutyric acid, valeric acid and isovaleric acid. In the intestine of healthy individuals, SCFAs are mainly acetic acid, propionic acid and butyric acid, among which butyric acid accounts for 95% of the total SCFAs content in the intestine (Hu et al., 2018). In recent years, many physiological and clinical studies have highlighted the health benefits of SCFAs, including improvements on weight management, glucose homeostasis, and the blood lipid profile (Kasubuchi et al., 2015). Butyric acid inhibits hepatic lipid accumulation mainly by focusing its targets on enzymes (e.g., FAS) and genes (e.g., acetyl-CoA carboxylase, ACC) related to fatty acid synthesis (Yao et al., 2022). Acetic acid regulates lipid metabolism mainly by modulating hepatic metabolic signaling pathways, such as activating PPAR-α signaling pathway, promoting fatty acids into mitochondria for β-oxidation, and regulating intracellular signal transduction related to cholesterol metabolism (Yamashita et al., 2007). Propionic acid has a unique role in weight control and intestinal hormones, modulating the levels of glucose-dependent insulinotropic polypeptide, insulin and amylin in mouse plasma, thereby reducing blood glucose and lipid accumulation in the blood (Gao et al., 2009). Zhao et al. (2024) found that cynaroside and other active components in Hazel Leaf polyphenolic Extract played anti-obesity effects by regulating intestinal flora, SCFAs and lipid metabolism. The research results showed that hazelnut leaf polyphenol extract significantly increased the intestinal microbial diversity and decreased F/B values in HFD fed mice, elevated the intestinal content of SCFAs, reduced the expression of lipid synthesis-related proteins SREBP1c, PPARγ and CCAAT/enhancer-binding protein alpha (C/EBPα), and promoted the phosphorylation level of adenosine 5′-monophosphate (AMP)-activated protein kinase (AMPK). The above phenomenon was also observed in animal experiments using cynaroside compounds alone by Yang et al. (2025). Their findings indicated that cynaroside upregulated uncoupling protein 1 (UCP-1), PPAR-α, CYP7A1 and carnitine palmitoyltransferase I (CPT-1) protein expression, and downregulated C/EBPα, PPARγ and fatty-acid synthase (FASN) proteins to regulate lipid metabolism. The intervention improved the diversity and composition of the gut microbiota by increasing the abundance of beneficial bacteria (e.g., *Akkermansia* and *Coriobacteriaceae*_UCG-002) and reducing the abundance of harmful bacteria (e.g., Lachnospiraceae) (Yang et al., 2025).

### 4.2 Antidiabetic activity of cynaroside

#### 4.2.1 Inhibiting the α-glucosidase and α-amylase activities

Glucose is the end product of two enzymes catalyzing carbohydrates. And α-amylase catalyzes the hydrolysis of polysaccharides such as starch, while α-glucosidase catalyzes the hydrolysis of oligosaccharides to produce glucose as the final step in carbohydrate digestion. Therefore, glucose uptake in the blood decreases when the carbohydrate hydrolases (α-amylase and α-glucosidase) lose their functions (Usman et al., 2019).

Kim et al. (2000) firstly found that cynaroside effectively inhibited postprandial hyperglycemia in non-insulin-dependent diabetic patients, with a mechanism closely related with the activities of α-glucosidase and α-amylase. The investigators used yeast α-glucosidase and porcine pancreatic α-amylase to determine the inhibitory activity by measuring the absorbance of the compound at 540 nm and 405 nm. Asghari et al. (2015) further investigated the hypoglycemic effect of each flavonoid component in *Salvia chloroleuca* Rech. f. & Aellen extract and plotted the percent inhibition versus concentration curve to determine half-maximal inhibitory concentration (IC50) values. The results indicated that the isolated cynaroside in the extract showed only a moderate inhibition of α-amylase (IC50 = 81.7 µM) and more significant inhibition of α-glucosidase (IC50 = 18.3 µM), and the positive control of acarbose (IC50 = 16.1 µM). Where the hydroxyl substitution in the B ring of the compound and the sugar moiety in the A ring are effective factors for the inhibitory activity of flavonoids. Orhan et al. (2016) supplemented relevant animal experiments to establish a streptozotocin (STZ) induced diabetic rat model to evaluate the antidiabetic effects of *Bidens tripartita* L. extract and to quantify the active components of the extract. The extract reduced blood glucose at 500 mg/kg, and the main active components in the extract were chlorogenic acid, luteolin and cynaroside. These studies provide the scientific basis for the development of new diabetes treatments, and cynaroside as a natural flavonoid holds promise as an effective drug for diabetes therapy, especially for diabetic patients with higher postprandial glucose. But its effect and safety in human body still need to be further studied.

#### 4.2.2 Increasing hepatic glycogen and muscle glycogen reserves

The liver plays an important role in glucose homeostasis by effectively transforming molecules into glycogen for storage, which absorbs about 35% of postprandial glucose. Glucose-6-phosphatase (G-6-Pase) is one of the rate-limiting enzymes in the liver to regulate glucose metabolism (Moore et al., 2012). Through inhibiting hepatic G-6-Pase activity, cynaroside increases the content of hepatic glycogen and reduced the breakdown of the phosphoryl acid of glycogen to glucose (Tan et al., 2025).

The glucose transporter type 1 (GLUT-1, non-insulin responsiveness) and glucose transporter type 4 (GLUT-4, insulin responsiveness) are two major glucose transporter proteins that regulate glucose uptake into various tissues. Among them, GLUT-1 is widely expressed, while GLUT-4 is mainly expressed in skeletal muscle and adipocytes (Sivitz et al., 1989). Cynaroside was reported to stimulate the skeletal muscle GLUT-4 expression and glucose uptake through the activation of the transcriptional activity of PPARγ pathway. PPARs, a nuclear receptor protein group, are transcription factors that play an important role in lipid metabolism and glucose homeostasis. There are three PPAR isoforms: α, β/δ, and γ (Hsia et al., 2010). Ong et al. (2011) studied plant Vernonia amygdalin had a protective effect on pancreatic β cells, increased the expression of GLUT-4 in rat skeletal muscle, promoted the translocation of GLUT-4 to the cell membrane, and also inhibited the activity of G-6-Pase. These are all related to the fact that the ethanol extract of *Vernonia amygdalina* Delile containing high levels of polyphenols, in which cynaroside is the main substance exerting the biological activity. Shin et al. (2013) found that the ethanol extract of *Prunus mume* (Siebold & Zucc.) could stimulate glucose uptake in C2C12 myotube cells (a muscle cell model) by regulating PPARγ. The main active component of *P*. *mume* by hyperliquid chromatography (HPLC) analysis is flavonoid compounds, such as cynaroside. *In vivo*, 5% ethanol extract of *P*. *mume* significantly reduced weight gain and fat accumulation caused by HFD, and improved fasting glucose levels and glucose tolerance in a HFD-induced obesity mouse model. *In vitro*, 400 and 800 μg/mL ethanol extract of *P*. *mume* significantly increased glucose uptake and PPARγ transcriptional activity in C2C12 myotubes, suggesting that cynaroside promotes glucose uptake through activation of the PPARγ pathway.

#### 4.2.3 Inhibition of dipeptidyl peptidase IV activity

Dipeptidyl peptidase IV (DPP-IV) rapidly inactivates the incretin hormones glucagon-like peptide-1 (GLP-1) and glucose-dependent insulinotropic polypeptide (GIP). Inhibition of DPP-IV prolongs and enhances the activity of endogenous GLP-1 and GIP, which serve as important prandial stimulators of insulin secretion and regulators of blood glucose control (Green et al., 2006). DPP-IV enzyme inhibitors have been shown to reduce hyperglycemia, improve impaired glucose metabolism, and promote insulin secretion by targeting pancreatic cells (Mu et al., 2006). Bansal et al. (2012) established a mouse model of HFD/STZ-induced diabetes to study the antidiabetic, anti-hyperlipidemic and antioxidant effects of flavonoids-rich extract from *Pilea microphylla* (L.) Liebm. The *in vitro* findings indicated that *P*. *microphylla* inhibited the DPP-IV enzyme in a dose-dependent manner. Besides, oral glucose tolerance test (OGTT) of lean mice showed that *P*. *microphylla* (600 and 900 mg/kg) dose-dependently reduced glucose fluctuation (AUC 0–120 min), suggesting the inhibition of DPP-IV. The main active components of *P*. *microphylla* were identified by HPLC analysis, including chlorogenic acid, cynaroside, etc.

### 4.3 Anti-inflammatory effect of cynaroside

**FIGURE 4:**
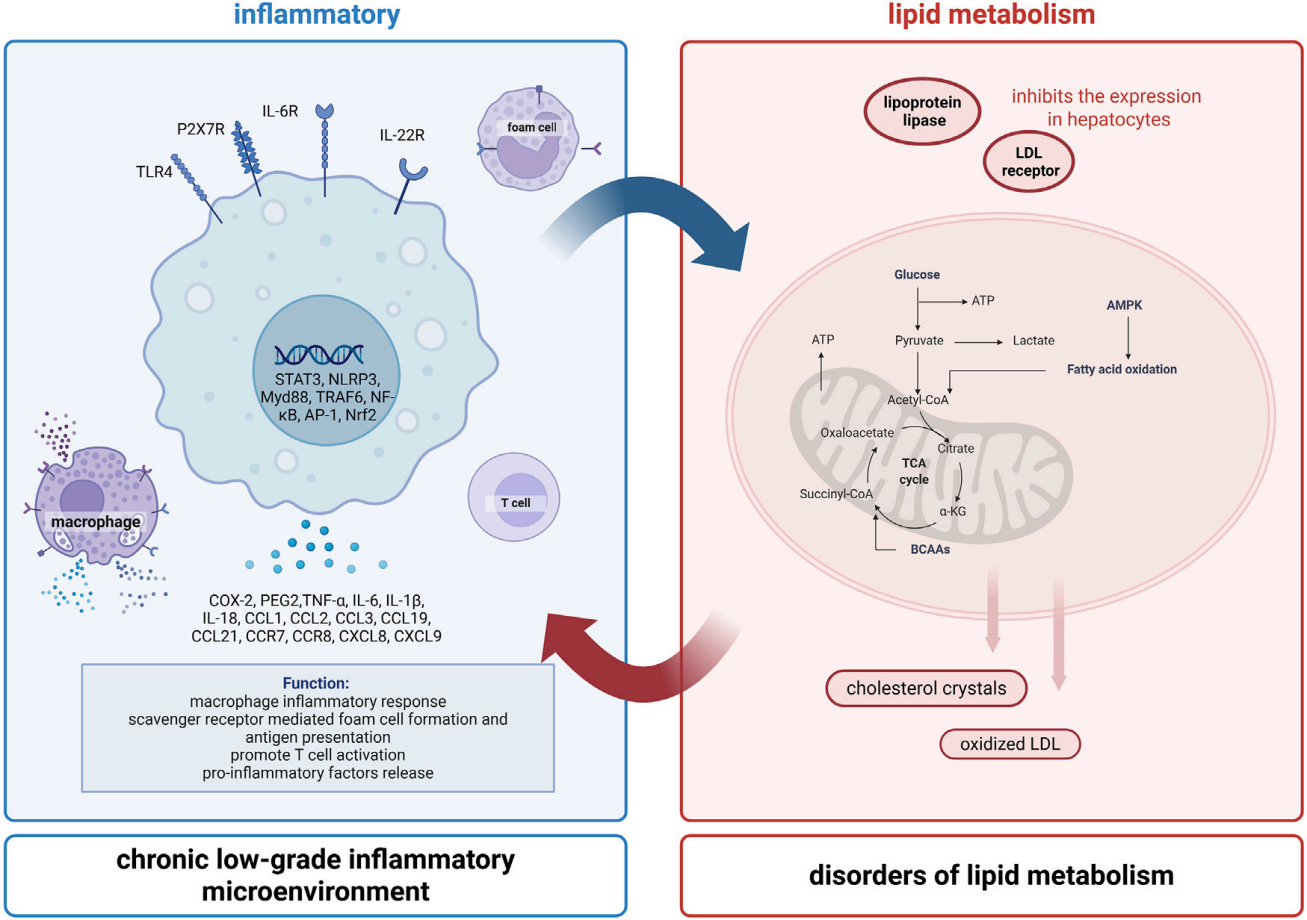
The interaction between inflammation and lipid metabolism. Inflammation and lipid metabolism disorders form a vicious circle through immune-metabolic interactions, which jointly drive the pathological process of many chronic diseases.

Inflammation and lipid metabolism disorders form a vicious circle through immune-metabolic interactions, which jointly drive the pathological process of many chronic diseases. The interaction mechanism of inflammation and lipid metabolism is shown in Figure 4. In inflammatory conditions, pro-inflammatory factors, such as tumor necrosis factor alpha (TNF-α) and interleukin-6 (IL-6) inhibit lipoprotein lipase activity and LDL receptor expression in hepatocytes, resulting in impaired TG breakdown, reduced LDL-C clearance and increased VLDL synthesis (Saltiel and Olefsky, 2017). Lipid disorders can also further activate the immune system. Free fatty acids (FFA) trigger macrophage inflammatory response through toll-like receptor 4 (TLR4)/nod-like receptor protein 3 (NLRP3) inflammasome pathway. And cholesterol crystals and oxidized LDL (ox-LDL) promote T cell activation and pro-inflammatory factors release through scavenger receptor mediated foam cell formation and antigen presentation, forming a chronic low-grade inflammatory microenvironment (Ouchi et al., 2011; Kawai et al., 2021).

**FIGURE 5:**
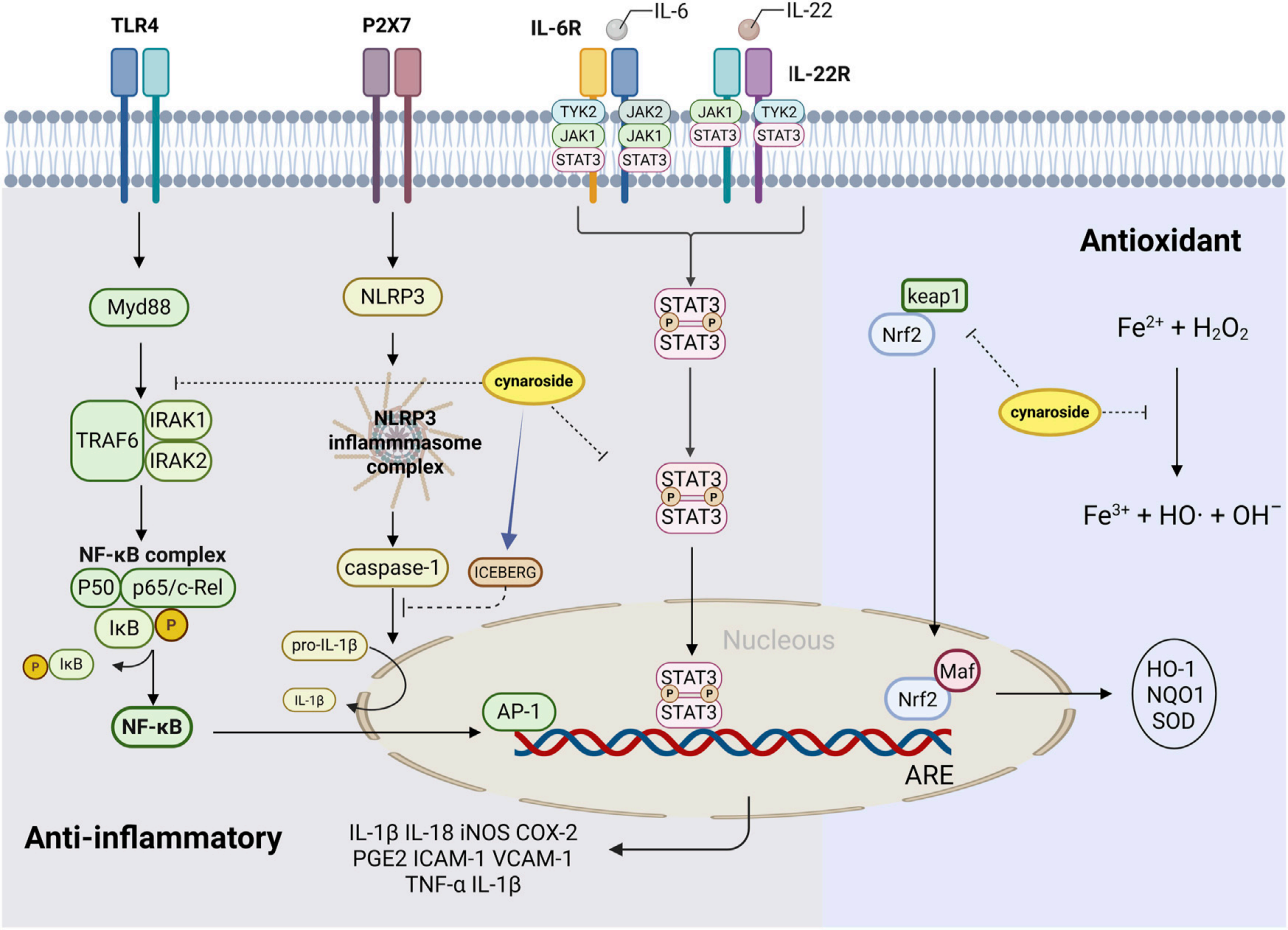
Antioxidant and anti-inflammatory effects of cynaroside. Regarding antioxidant effect, cynaroside increased HO-1 activity and reduced ROS and lipid peroxidation by upregulating Nrf2, and favored Fe^2+^ release. In relation to anti-inflammatory effects, cynaroside effectively inhibited the production of NO and PGE2 and inhibited the expression of the corresponding enzymes, like iNOS and COX-2, which was associated with downregulation of the TLR4/Myd88/NF-κB/AP-1 pathway. Cynaroside blocks IL-1β secretion and inhibits the activation of NLRP3 inflammasome by increasing ICEBERG (caspase-1 inhibitor) levels. Cynaroside inhibits the expression of inflammation-related target genes by inhibiting the damage of STAT3 nuclear translocation, thereby antagonizing the JAK/STAT3 pathway activated by IL-22 and IL-6 and reducing the expression of inflammation molecules such as ICAM-1, VCAM-1, TNF-α, IL-1β and COX-2.

Numerous studies have shown the strong anti-inflammatory properties of cynaroside isolated from various plants, consolidating its role as a promising therapeutic agent. Table 1 presents the key studies exploring the underlying mechanisms of these activities of cynaroside. The modulation of involved signaling pathways is summarized in Figure 5. Cynaroside not only blocks the interaction between ligands (pathogen-associated molecular patterns, PAMPs) and their receptor (pattern recognition receptors, PRRs), but also inhibits the activation of downstream signaling nuclear factor kappa-B (NF-κB), NLRP3 and janus kinase/signal transducer and activator of transcription (JAK/STAT) inflammatory pathways (Surh et al., 2001; Fujioka et al., 2004; Bouyahya et al., 2023).

**TABLE 1:** Antioxidant and anti-inflammatory effects of cynaroside.

Origins	Experimental model	Dose/Duration	Efficacy	Mechanism	References
Carthamus lanatus L	Human neutrophils	1 mg/30 min	Anti-inflammatory	↓ROS	Jalil et al. (2003)
—	Human endothelial cells	20 µM/48 h	Anti-inflammatory, antioxidant	↓CCL1, CCL2, CCL3, CCR7, CCL19, CCL21, CCR8, CXCL12 ↑IL10-RB, ICEBERG	De Stefano et al. (2021)
—	RAW 264.7 cells	5, 10, 25, 50 µM/2 h	Anti-inflammatory	↓PGE2, iNOS, COX-2, NF-κB, AP-1	Park and Song (2013)
Vernonia amygdalina leaves	Swiss albino mice	30, 80, 800 mg/kg	Anti-inflammatory	↓significant reduced inflammation and paw edema	Canh Pham et al. (2024)
—	HEKn/Imiquimod mouse model	20 μM/48 h, 3 days, 6 days	Anti-inflammatory	↓Metabolism, lipid peroxidation, proliferation, KRT10, p-STAT3	Palombo et al. (2016)
—	Sprague-Dawley adult male rats	10, 20, 40 mg/kg/7 days	Anti-inflammatory	↓TNF-α, IL-1β, IL-18, CD68-positive cells, NLRP3, caspase 1 ↑ Cell viability	Lang et al. (2024)
—	Human dental pulp cells	1, 5, 10, 20, 30 µM/24, 48, 72 h	Anti-inflammatory	↓ICAM-1, VCAM-1, TNF-α, IL-1β, MMP-2, MMP-9, COX-2, p-JNK, ROS	Ji-Eun et al. (2024)
—	T.gondii-infected mice	0–200 mg/kg	Anti-inflammatory, antioxidant	↓TNF-α, IL-6, IL-1β, ROS ↓TLR4, Myd88, TRAF6, p-NF-κB p65 ↓P2X7R, NLRP3, caspase 1, IL-1β, IL-18 ↑SOD, GSH ↑Nrf2, HO-1, NQO-1, GCLC	Han et al. (2023)

Abbreviations: ↓, upregulation; ↑, downregulation; ROS, reactive oxygen species; CCL1, C-C motif chemokine ligand 1; CCL2, C-C motif chemokine ligand 2; CCL3, C-C motif chemokine ligand 3; CCR7, C-C chemokine receptor type 7; CCL19, C-C motif chemokine ligand 19; CCL21, C-C Motif Chemokine Ligand 21; CCR8, C-C chemokine receptor type 8; CXCL12, C-X-C motif chemokine ligand 12; IL10-RB, interleukin 10 receptor subunit beta; PGE2, prostaglandinE2; iNOS, inducible nitric oxide synthase; COX-2, cyclooxygenase-2; NF-κB, nuclear factor kappa-B; AP-1, activator protein-1; KRT10, Keratin 10; p-STAT3, phospho-signal transducer and activator of transcription 3,TNF-α, tumor necrosis factor-α; IL-1β, interleukin-1β; IL-18, interleukin-18; NLRP3, NOD-like receptor thermal protein domain associated protein 3; ICAM-1, intercellular cell adhesion molecule-1; VCAM-1, vascular cell adhesion molecule-1; MMP-2, matrix metallopeptidase 2; MMP-9, matrix metallopeptidase 9; p-JNK, phospho-c-Jun N-terminal kinases; IL-6, interleukin-6; TLR4, toll like receptors 4; Myd88, myeloid differentiation primary response protein 88; TRAF6, TNF; receptor associated factor 6; p-NF-κB, phospho-nuclear factor kappa-B,P2X7R, purinergic receptor P2X 7; GSH, glutathione; Nrf2, nuclear factor erythroid 2-related factor 2; HO-1, heme oxygenase 1; NQO-1, quinone oxidoreductase 1; GCLC, glutamate-cysteine ligase catalytic subunit.

NF-κB consists of p65 and p50, serving as transcription factors that regulate the expression of genes involved in inflammation, cell differentiation and proliferation (Surh et al., 2001). Activator protein-1 (AP-1) is a ubiquitous protein that exists in the cytoplasm as homodimer or heterodimer with the jun and fos families (Fujioka et al., 2004). The activation of NF-κB and AP-1 is highly correlated with the induction of inflammatory enzymes and is regulated by the phosphorylation of p65 and c-jun. Therefore, NF-κB and AP-1 are considered key therapeutic targets for suppressing inflammatory responses (Kim et al., 2013). Park and Song (2013) found that cynaroside effectively inhibited the production of nitric oxide (NO) and prostaglandin E2 (PGE2) and inhibited the expression of the corresponding enzymes, like inducible NO synthase (iNOS) and cyclooxygenase-2 (COX-2), which was associated with downregulation of the NF-κB/AP-1/PI3K-Akt pathway. *In vitro*, inhibition of lipopolysaccharide (LPS)-stimulated RAW 264.7 cells showed that cynaroside inhibited the production of NO and PGE2 by inhibiting p65 and c-jun translocation, suppressing the activation of NF-κB and AP-1, and reducing the protein expression of iNOS and COX-2 (Park and Song, 2013). Han et al. (2023) treated mice infected with *Toxoplasma gondii (T. gondii)* with different doses of cynaroside (0, 50, 100, 200 mg/kg for 7 days) and further detected the expression of key proteins in inflammatory and oxidative stress related pathways in the liver. The results showed that cynaroside significantly reduced inflammatory factors TNF-α, IL-6, and IL-1β, as well as the oxidative product malondialdehyde (MDA), while significantly increasing antioxidant enzymes superoxide dismutase (SOD) and glutathione (GSH). Moreover, key proteins in the TLR4/NF-κB pathway, including TLR4, myeloid differentiation primary response 88 (Myd88), TNF receptor associated factor 6 (TRAF6) and p-NF-κB p65, were all significantly downregulated in the mouse liver. This confirms that cynaroside can inhibit *T. gondii* induced liver injury by blocking inflammatory response and enhancing antioxidant capacity. TLR4 is a member of the toll-like receptor family, which can promote the activation of NF-κB signaling pathway (Peng et al., 2016).

The NLRP3 inflammasome is a multiprotein complex in the innate immune system. Its dysregulation can drive chronic inflammation in diseases, making it a key therapeutic target for modulating inflammatory pathways (Xu et al., 2024). Purinergic 2X7 Receptor (P2X7R) is an inflammatory receptor involved in the activation of inflammatory vesicles NLRP3, which can further upregulate the release of inflammatory factors, such as IL-18 and IL-1β (Han et al., 2018). Han et al. (2023) also verified that the expressions of key proteins in the P2X7R/NLRP3 pathway, including P2X7R, NLRP3, caspase 1, IL-1β and IL-18, were significantly reduced in the liver of rats infected with *T. gondii*. The results of Lang et al. (2024) consistently demonstrated that cynaroside could inhibit NLRP3 inflammasome activation in rats induced by methotrexate. The study used 7 mg/kg of methotrexate to treat Sprague-Dawley rats for 3 days, followed by treatment with different doses (10, 20, 40 mg/kg) of cynaroside for 7 days, to evaluate the protective potential of cynaroside against intestinal inflammation. The results showed that administration of 20 or 40 mg/kg of cynaroside reduced inflammatory cell infiltration in the intestines induced by methotrexate, increased the number of goblet cells, decreased serum levels of TNF-α, IL-1β, and IL-18, as well as the rate of CD68-positive cells. In addition, cynaroside increases ICEBERG (caspase-1 inhibitor) levels by blocking IL-1β secretion and suppressing the activation of the NLRP3 inflammasome.

During the process of vascular inflammation, the activation of the JAK/STAT3 pathway is a key mechanism for regulating endothelial cell inflammation, migration, and proliferation of vascular smooth muscle cells (VSMCs) (Zegeye et al., 2018). IL-6 stimulates JAK and promotes gp130 phosphorylation of JAK, which induces the phosphorylation and dimerization of STAT3. STAT3 then translocates to the nucleus to activate the expression of pro-inflammatory genes (Moshapa et al., 2019). It is important to note that it has been demonstrated in keratinocytes that cynaroside can downregulate inflammation-related target genes by inhibiting the damage of STAT3 nuclear translocation, which counteracts JAK/STAT3 pathway activated by IL-22 and IL-6 (Palombo et al., 2016). De Stefano et al. (2021) elucidated that cynaroside exerted antiproliferative effects by inhibiting the STAT3 pathway in human umbilical vein endothelial cells (HUVEC), and down-regulating the transcription of IL-1β, IL-6 and TNF-α, thereby exerting antioxidant and anti-inflammatory effects. Using cultured HUVEC, cynaroside was treated at a concentration of 20 µM for 48 h, and RNA was extracted and analyzed for many genes in inflammatory pathways. Cynaroside interventions significantly inhibited a variety of cytokines and related receptors involved in the inflammatory pathway, the chemokines and their receptors that were downregulated included C-C motif ligand (CCL)1-3, CCL19, CCL21, C-C motif receptor (CCR)7, CCR8, C-X-C motif chemokine ligand (CXCL)2, CXCL8, CXCL9 and CXCL12. CCL1 is a T-cell chemokine that mediates monocyte chemotaxis and immune regulation. CCR8 is a key transmembrane receptor for inflammatory cell migration that interacts with CCL1 (Haque et al., 2001). Parallel downregulation patterns are observed in CCL11 (eosinophil chemoattractant via CCR3) and CCL3 (macrophage-activating cytokine), both key mediators of inflammatory cascades (Kindstedt et al., 2017; Li et al., 2024). Cynaroside similarly suppresses CXCL12, a multifunctional chemokine involved in lymphocyte/monocyte recruitment, tumor surveillance, and metastasis regulation (Conley-LaComb et al., 2016). Leukotriene B4 receptor (LTB4R) is a lipid mediator receptor in metabolism pathways of arachidonic acid, and IL-1β is a central inflammatory cytokine (Libby, 2017). Their reduction further confirms the broad anti-inflammatory effects of cynaroside. Palombo et al. (2016) found that in the imiquimod murine model of psoriasis, cynaroside treatment blocked the nuclear translocation of phosphorylated STAT3 induced by the stimulation of proinflammatory cytokines IL-22 and IL-6, thus exerting anti-proliferative and anti-inflammatory effects.

Ji-Eun et al. (2024) further demonstrated that cynaroside reduced the expression of inflammatory molecules such as ICAM-1, VCAM-1, TNF-α, IL-1β and COX-2, and reduced reactive oxygen species (ROS) formation by inhibiting the activation of p-JNK in the MAPK pathway, thus exerting a protective effect against inflammation and oxidative stress in methylglyoxal-induced human dental pulp cells. Canh Pham et al. (2024) also extracted cynaroside from *V. amygdalina* leaf and found that cynaroside had a very high affinity (>10 kcal/mol) for iNOS and COX-2. Jalil et al. (2003) confirmed the anti-inflammatory activity of cynaroside *in vitro* by studying the inhibitory effect of four total extracts (dichloromethane, methanol, 50% methanol, water extract) and their main active ingredients (cynaroside) on human neutrophils induced by the aerial parts of *Carthamus lanatus* L.

### 4.4 Antioxidant effect of cynaroside

Oxidative damage is undeniably responsible for lipid-related metabolic disease progression. Intracellular oxidative stress is caused by the imbalance between the level of ROS and antioxidant defense. If excessive ROS is produced by oxygen enzymes along the respiratory chain, it will cause DNA damage, protein crosslinking, lipid peroxidation of polyunsaturated fatty acids and further lead to cell aging, cell membrane damage, etc. (Barrera, 2012). In many cases, inflammation and oxidative stress are mutually promoted and activated. Amounts of studies also have demonstrated the strong antioxidant properties of cynaroside (Table 1; Figure 5).

Dong et al. (2023) first found that cynaroside as a flavonoid compound had *in vitro* antioxidant capacity and delayed the oxidation of LDL in a dose-dependent manner. The antioxidant activity is partly related to its flavonoids, which are chelators of hydrogen donors and metal ions. It can chelate Fe^2+^ through its C ring, thus avoiding the formation of the most harmful ROS (OH-). And Fe^2+^ is the catalyst needed to produce OH- radical in Fenton reaction and participates in ferroptosis (Dong et al., 2023).

Nuclear factor erythroid 2-related factor 2 (Nrf2) has been shown to alleviate liver oxidative damage and cell apoptosis by upregulating the expression of antioxidant and detoxification pathways. Han et al. (2023) found that after treating *T. gondii*-infected mice with cynaroside, the expressions of key proteins in the Nrf2/heme oxygenase-1 (HO-1) antioxidant pathway, including Nrf2, HO-1, NAD(P)H quinone oxidoreductase-1 (NQO-1) and glutamate-cysteine ligase (GCLC), were significantly increased. After treatment with cynaroside at doses of 100 mg/kg and 200 mg/kg, the expressions of these oxidative stress inhibitors were significantly upregulated. Moreover, after treatment with cynaroside at a dose of 50 mg/kg, the expressions of Nrf2, HO-1 and NQO-1 were also increased significantly. The activation of Nrf2/HO-1 pathway to protect cells from the harmful effects of oxidative stress is an adaptive response. When ROS increase, Nrf2 is activated and rapidly triggers the expression of antioxidant enzymes HO-1 and NQO-1 to exert cellular antioxidant functions (Xu et al., 2017).

De Stefano et al. (2021) further explored that cynaroside affected the cholesterol hydroxylation pathway, increased cholesterol levels and substantially reduced the levels of 7-α-hydroxycholesterol, 7-β-hydroxycholesterol and 7-ketocholesterol in the human keratinocyte model. These hydroxycholesterol is often found to be increased in many pathological situations, causing cytotoxic effects by inducing oxidative stress and dysfunction of organelles, such as mitochondria, lysosomes and peroxisomomes. In addition, cynaroside regulated the fatty acid hydroxylation pathway, leading to increased linoleic acid content and decreased contents of 2-hydroxypalmitate, 2-hydroxystearate and 2-hydroxydecanoate.

## 5 The effects of cynaroside on lipid-related diseases

### 5.1 Obesity

Obesity is a chronic metabolic syndrome with energy metabolism imbalance, characterized by abnormal accumulation of adipose tissue and glucolipoprotein metabolism (Schwab et al., 2014). The imbalance caused by obesity can lead to T2DM, MASLD, atherosclerotic cardiovascular disease, nephrotic syndrome and other chronic diseases (Li et al., 2023). According to the Global Burden of Disease Report released by the World Health Organization (WHO), as of 2023, 34.8% of the global adult population is overweight (BMI ≥ 25 kg/m^2^) and 14.1% meets the diagnostic criteria for obesity (BMI ≥ 30 kg/m^2^) (NCD Risk Factor Collaboration, 2024). Longitudinal epidemiological surveys revealed that the global adult obese population more than doubled between 1990 and 2022. In 2022, the total number exceeded 1 billion, accounting for about 12.5% of the global population (Jaacks et al., 2019). Clinical treatment of obesity is based on lifestyle improvement, including dietary structure modification and exercise therapy (Medina-Remón et al., 2018). However, the sustainability of behavioral interventions is often limited by patients’ compliance, which is prone to weight regain. In terms of drug treatment, orlistat (a pancreatic lipase inhibitor) reduces energy intake by reducing dietary fat absorption, while GLP-1 receptor agonists (such as selmegallutide) exert weight-loss effects through central appetite suppression and peripheral metabolic regulation (Perdomo et al., 2023). In addition, recently, the role of the gut microbe-host metabolic axis in obesity has become increasingly clear. The abundance changes of specific bacterial groups can affect SCFAs synthesis, bile acid metabolism, and intestinal barrier integrity, thus interfering in energy absorption and storage efficiency. For example, phenolamide compounds isolated from apricot pollen can ameliorate obesity-related inflammation by regulating gut flora composition (e.g., increasing the abundance of *Bifidobacterium* and *Lactobacillus*) and repairing abnormal metabolite profiles (Zhang et al., 2023). Fermented Tartary buckwheat extract showed dual regulatory potential, which could not only enhance glucose and lipid homeostasis, but also inhibit the release of proinflammatory factors and reverse hepatocyte steatosis (Yang et al., 2023).

Yang et al. (2025) established a obesity model fed by 1 week HFD in 6-week-old male C57BL/6J mice, and simultaneously gave them low dose (5 mg/kg), medium dose (50 mg/kg) and high dose (100 mg/kg) of cynaroside for 12 weeks. In the HFD group, the body weight, liver weight, adipose tissue weight of mice were significantly increased. In addition, obese mice had impaired glucose tolerance and increased steatosis and damage in hepatocytes. Cynaroside intervention significantly improved these phenomena, suppressed mouse weight gain, reduced TC, TG and LDL-C levels in serum, inhibited adipocyte hypertrophy and reduced the level of inflammatory factors (Yang et al., 2025). Gebhardt (1998) found that cynaroside, the active ingredient in *C. scolymus* extract, inhibited cholesterol biosynthesis in hepatocytes by inhibiting HMG-CoA activity through *in vitro* cell experiments. Azevedo et al. (2010) supplemented the relevant animal experiments. In their study, six-week-old healthy male Wistar rats were treated with 2 mg/kg ursolic acid or cynaroside for 7 consecutive days. By collecting and testing rat blood and liver samples, cynaroside was found to reduce TC and LDL-C levels. Ong et al. (2011) researched the effects of polyphenols-rich *V. amygdalina* extracts in a rat model of streptozotocin-induced diabetes and found that extract treatment not only reduced blood glucose and significantly reduced TG and TC levels in the serum of rats (18.2% and 41%, respectively). Analysis of the chemical composition of the extract using HPLC found that the active ingredient playing the main role was also cynaroside. Most of these studies have their limitations. Gebhardt et al. first found the lipid-lowering effect of cynaroside, but they gave a complex plant extract and did not detail the proportion of active ingredients. Azevedo et al. used a single component and supplemented animal experiments, but the number of animals used in this study was relatively small and the duration of the experiment was short to fully assess the long-term effects of cynaroside on lipid profiles. The study did not explore the exact pathway by which cynaroside affects lipid metabolism, nor did it compare it with existing anti-diabetic drugs. In contrast, Yang et al. conducted a more comprehensive study by using a single component for a longer period of time in animal experiments, comparing it with existing drugs to explore its potential mechanisms, and comparing the effects of cynaroside on insulin sensitivity or glucose tolerance tests and other organs.

Cynaroside can simulate endogenous estrogen and play an estrogen-like role to improve blood lipid profile, thus improving obesity. Ammar et al. (2016) established an ovariectomized rat model to explore the estrogenic activity of cynaroside. The animal experiment used 24 female Sprague Dawley rats weighing 100–140 g and randomized them into four groups with following treatments: normal control, bilateral oophorectomy, 17-β-estradiol (10 μg/kg body weight) and cynaroside (5 mg/kg body weight). Cynaroside was found to significantly reduce the TC, TG, LDL-C and TC/HDL-C ratio, and significantly increase the HDL-C level. At a dose of 5 mg/kg, it showed significant estrogenic activity and maintained normal uterine weight and plasma estradiol levels. Cynaroside significantly inhibited bone conversion markers such as bone-specific alkaline phosphatase (BALP), plasma osteocalcin (OCN), type I procollagen N-terminal peptide (PINP) and type II collagen C-terminal peptide (CTX-II).

### 5.2 Metabolic dysfunction-associated steatotic liver disease

MASLD has emerged as a prominent global health challenge due to its asymptomatic manifestations, high prevalence and potential intra and extrahepatic consequences, often concurrent with the prevalence of obesity and T2DM and is a major cause of liver-related morbidity and mortality (Anonymous, 2025). It is characterized by excessive hepatic lipid accumulation (liver steatosis), which can lead to inflammation (steatohepatitis) and progressive fibrosis (European Association for the Study of the Liver (EASL) et al., 2024). By 2021, the global prevalence of MASLD has reached 32.4% and shows a continuous upward trend (Tincopa and Loomba, 2024). This disease spectrum covers stages from early simple hepatic steatosis to metabolic-associated steatohepatitis (MASH), which can further progress to hepatocirrhosis and hepatocellular carcinoma (HCC) (Tincopa and Loomba, 2024). The severity of fatty liver disease is influenced by multiple factors, including genetic susceptibility, nutritional composition, obesity, insulin resistance, gut microbiome and a range of disease endocrine effectors. For the treatment of MASLD patients, it is recommended to base the approach on lifestyle changes and enhance the management of comorbidities, including weight loss, dietary modifications, physical exercise, and abstinence from alcohol. Resmetirom has histological efficacy for steatohepatitis and liver fibrosis with acceptable safety and tolerability. Currently, there are no targeted drug therapies for MASH in the cirrhosis stage, the treatment principles are similar to those for cirrhosis caused by other reasons, including metabolic adjustment medications, nutritional counseling, monitoring of portal hypertension and HCC, as well as liver transplantation for decompensated cirrhosis. Recent studies have found that cynaroside has a good improvement effect on MASLD, which has been confirmed through *in vitro* and *in vivo* experiments.

Yixiao et al. (2023) established a mouse model of HFD-induced MASLD in eight-week-old male C57BL/6 mice, and the groups were control group, HFD group, low dose Sanren Tang (crude drug 1.09 g/mL, 20 mL/kg body weight) group, high dose Sanren Tang (crude drug 2.18 g/mL, 20 mL/kg body weight) group and obe cholic acid (10 mg/kg) group. The animal study induced MASLD in mice by a 13-week HFD, followed by 3 weeks of intervention with Sanren Tang (a characteristic component of Herba Lophatheri) and obe cholic acid. They also verified that cynaroside as the main active component of Sanren Tang improved liver histology and fat deposition in MASLD mice, decreased insulin resistance index, NAFLD activity score (NAS) and serum alanine aminotransferase (ALT) and liver TG levels. RNA sequencing was performed and the liver transcriptome was analyzed, revealing that this improvement was associated with retinol metabolism, cytokine-cytokine receptor interaction, and PPARγ signaling. This study is a controlled study with effective control of experimental variables, but there are still some limitations. The relatively small sample size of the study, and the complex identification of SRT components, may not fully explain all of its pharmacological effects, which may limit the generalizability of the results.

Meng et al. (2023) evaluated the efficacy of cynaroside in liver fibrosis in a HFD-induced MASH mouse model. The model was developed by inducing lipotoxic liver injury in BKS-db males mice (Jingle Biotech) fed by SYGR01 HFD (4.13 kcal/g) for 4 weeks, and the mice were divided into blank control group, MASH model group, cynaroside (20 mg/kg) group and positive control pioglitazone group. The results showed that cynaroside alleviated liver steatosis (45% less oil red staining area) and reduced F4/80 marked inflammatory cell infiltration (60% less than model group). In addition, Meng et al. verified the role of cynaroside in the regulation of fibroblast growth factor receptor 2 (FGFR2)/TGF-β signaling cascade in the development of liver fibrosis (Meng et al., 2023). Cynaroside blocked the activation and development of liver fibrosis by inhibiting the overexpression of FGFR2 and the excess of basic fibroblast growth factor (bFGF), thus reducing hepatic stellate cell (HSC) activation and collagen secretion in hepatocytes (Sato-Matsubara et al., 2017; Wang et al., 2020). In addition, cynaroside reduced α-SMA (45% reduction) and Collagen I (50% reduction) expression in LX-2 cells and truncated the paracrine activation loop between HSC and hepatocytes (Meng et al., 2023). The study design is reasonable and comprehensive, including both cell experiments and animal experiments as well as molecular docking and molecular dynamics simulation. However, there is a lack of comprehensive molecular-level explanation for the mechanism of cynaroside in treating liver fibrosis. Although cynaroside has shown inhibitory effect on FGFR2 *in vitro* and animal models, its pharmacokinetic and pharmacodynamic characteristics in human body need to be further studied.

Jia et al. (2022) found that Mailuoning oral liquid (7.8 mL/kg or 23.4 mL/kg, for 4 weeks) effectively alleviated lipid deposition by regulating PGC-1α/PPARα signaling pathway in MASLD by using methionine and choline deficient (MCD) diet-induced MASH for 2 weeks in mice. PPARα is a member of the ligand-induced transcription factor nuclear receptor family, crucial for regulating various metabolic pathways, including lipid metabolism, bile acid and cholesterol metabolism, as well as inflammation. The transcriptional activation of PPARα is regulated by multiple co-activators, with PGC-1α being the primary reported co-activator involved in activating PPARα. In this study, Mailuoning oral liquid increased the nuclear accumulation of PPARα and the expression of PGC-1α in the livers of MCD diet-fed mice, and also enhanced the expression of multiple downstream genes of PPARα (Pawlak et al., 2015). Through HPLC, cynaroside was found to be the active component of Mailuoning oral liquid that significantly reduced lipid accumulation in hepatocytes stimulated by non-esterified fatty acids (Jia et al., 2022).

Zhu et al. (2021) established an *in vitro* model of MASH induced by palmitic acid (PA) and found that cynaroside could stimulate hepatocyte regeneration by activating STAT3 pathway. In their study, LO2 cells (human hepatocyte cell line) and primary hepatocytes isolated from C57BL/6J mice were treated with PA (500 μM) for 48 h to induce steatosis. Upon addition of cynaroside (20 μM), the PA induced proliferation inhibition was reversed (MTT absorbance returned to 85% of control), and the STAT3 pathway increased cyclin D1 and c-myc by increasing phospho-STAT3 levels 3.2-fold (Zhu et al., 2021). The cyclin D1 and c-myc positively regulated cell cycle progression and decreased the expression of the cell cycle progression inhibitor p21 (Lu et al., 2018). Musolino et al. (2022) also demonstrated the potential anti-inflammatory and reduced lipid accumulation of *O. europaea* L. folium using an *in vitro* model of MASH. The main polyphenolic compounds in the *Olea europaea* leaf extract include cynaroside and oleuropepein (Guinda et al., 2015). A rat liver tumor cell line (McA-RH7777) was exposed to oleic acid to mimic the effects of lipid accumulation and inflammatory status. Using nile red and oil red O staining to detect lipid accumulation, and cytokines bioplex assay to assess inflammation, it was found that olive leaf extracts at concentrations of 25, 50 and 100 μg/mL were able to reduce intracellular lipid content and thereby combated the intracellular inflammatory state (Musolino et al., 2022). Neither study included *in vivo* experiments, and the cell models and experimental conditions used in the studies may not fully simulate the complex physiological environment *in vivo*.

### 5.3 Type 2 diabetes mellitus

Diabetes mellitus (DM) is a kind of chronic systemic disease with glucose metabolism disorders as the core, which is listed as a key disease for global public health prevention and control by the WHO (Evaluation I.f.H.M.a, 2024). According to its pathological characteristics, DM can be divided into type 1 diabetes (T1DM) and T2DM. T1DM is characterized by absolute insulin deficiency caused by the autoimmune destruction of islet β-cells, while T2DM is attributed to the relative insufficiency of insulin secretion and mainly caused by insulin resistance (Sun et al., 2022). In recent years, the global prevalence of T2DM has increased exponentially, and its disease burden is closely related with obesity, sedentary lifestyle and genetic susceptibility (Sun et al., 2022). T2DM is commonly associated with abnormal lipid metabolism and considered a major risk factor for premature development of atherosclerosis and cardiovascular complications (Saltiel and Kahn, 2001; Biddinger and Kahn, 2006). Long-term imbalance in blood glucose homeostasis can cause multiple organ damages through various metabolic routes. Microvascular complications include diabetic retinopathy, peripheral neuropathy and diabetic nephropathy. Macrovascular lesions significantly increase the risk of atherosclerosis, myocardial infarction and stroke (Singh et al., 2025). The treatment of T2DM is based on lifestyle intervention, combined with biguanides, sodium-dependent glucose transporters 2 (SGLT2) inhibitors, and GLP-1 receptor agonists to improve glycemic control. Recently, a number of studies have shown that natural ingredients such as cynaroside also have good hypoglycemic effects (Xu et al., 2018; Majety et al., 2023). Research of natural extracts and compounds containing cynaroside have shown potential in addressing diabetic symptoms and complications (Table 2).

**TABLE 2:** Antidiabetic effect of cynaroside.

Origins	Experimental model	Dose/Duration	Efficacy	Mechanism	References
Heliotropium procumbens Mill.	—	1.97 ± 0.14 mmol ACAE/g Methanol Extract	Antidiabetic	↓ α-glucosidase	Ozntamar-Pouloglou et al. (2023)
Ephedraceae leaf	Male Wistar rats	200 mg/kg/90 days	Antidiabetic	↓ α-amylase, pancreas and intestine lipase	Saidi et al. (2022)
Ethanolic Turnera subulata Sm. flower	Adult zebrafish	4, 20, 40 mg/kg	Antidiabetic, anti-inflammatory, antinociceptive	↓ α-glucosidase	Rebouças et al. (2022)
Bidens tripartita	Wistar Albino male rats	100 mg/kg/7 days	Antidiabetic, antioxidant	↓ α-glucosidase, α-amylase	Orhan et al. (2016)
Tephrosia humilis	—	—	Antidiabetic, antioxidant	↓ aldose/aldehyde reductase enzymes	Plioukas et al. (2016)
Salvia chloroleuca	—	12.5, 25, 50, 100, 150 µM/30 min	Antidiabetic	↓ α-glucosidase, α-amylase	Asghari et al. (2015)
Prunus mume fruits	C2C12 myotubes	5% ethanol extract of Prunus mume fruits	Antidiabetic	↑PPAR-γ mRNA	Shin et al. (2013)
Vernonia amygdalina Del.	STZ—induced diabetic rats	400 mg/kg/28 days	antidiabetic	↓ glucose-6-phosphatase ↓ triglyceride, total cholesterol ↑ GLUT 4 translocation	Ong et al. (2011)

Abbreviations: ↓, upregulation; ↑, downregulation; GLUT 4, glucose transporter type 4; PPARγ, peroxisome proliferators-activated receptors γ; mRNA, messenger Ribonucleic acid; ACAE, acarbose equivalent.

Cynaroside effectively reduces postprandial hyperglycemia in T2DM mice by inhibiting the activity of α-glucosidase and α-amylase (Kim et al., 2000; Asghari et al., 2015). The hydroxyl substitution in the B ring of the compound and the sugar moiety in the A ring are effective factors for the inhibitory activity of flavonoids. Then Orhan et al. confirmed this view with a STZ-induced diabetes model (Orhan et al., 2016). Shojaeifard et al. (2023) selected 50 different samples of 32 *Salvia* species and measured their inhibitory activity against α-glucosidase at three different concentrations (250, 500, and 1,000 μg/mL) and compared them with the positive control drug acarbose. Research has found that the inhibitory activities of *Salvia multicaulis, Salvia santolinifolia, Salvia dracocephaloides, and Salvia eremophila* were stronger than those of acarbose (*p* < 0.05), with their IC50 values ranging from 26.23 to 92.35 μg/mL. Through phytochemical analysis, eight common α-glucosidase inhibitors were isolated, including cynaroside, luteolin-7-O-glucuronide, apigenin-7-O-glucoside, apigenin-7-O-glucuronide, hispidulin-7-O-glucuronide, hispidulin-7-O-glucoside, rosmarinic acid and carnosic acid. The content of these compounds in active species ranges from 1.5% to 95.0%, with cynaroside detectable in most α-glucosidase inhibitor species.

Cynaroside increases liver glycogen content by inhibiting liver G-6-Pase activity, and can also stimulate skeletal muscle GLUT4 expression and glucose uptake by activating PPARγ pathway and increase muscle glycogen content. Ong et al. (2011) established a single dose of STZ (65 mg/kg, for 5 days) induced diabetic animal model and identified cynaroside as the active ingredient in the ethanol extract of *V*. *amygdalina* STZ induced diabetic rats were randomly selected and categorized into three groups (vehicle, 500 mg/kg metformin and 400 mg/kg *V amygdalina*) for a 28-day chronic study monitoring body weight, food and water intake, detecting insulin, TG, TC levels and hepatic G-6-Pase activity. The experimental results showed that fasting blood glucose decreased significantly after 28 days of treatment (32.1%), histology showed less pancreatic cell damage (no vacuoles and less granulation), more functional cells, GLUT4 expression in skeletal muscle (24%) and transposition to the plasma membrane (35.7%), and inhibited the activity of key hepatic gluconeogenic enzyme G-6-Pase (40% inhibition). This conclusion was also confirmed experimentally by Ighodaro and Akinloye (2017). Similarly, diabetes was induced in experimental animals via single intraperitoneal dose (55 mg/kg) of freshly prepared STZ. *Sapium ellipticum* was evaluated at 400 and 800 mg/kg of body weight against metformin (12 mg/kg). Treatments were done orally, twice daily at 8 h interval for a period of 21 days. Studies of the active ingredient cynaroside of *S. ellipticum* leaf extract revealed that *S. ellipticum* significantly reduced fasting blood glucose levels by 46.5% and 44.4% (doses of 400 and 800 mg, respectively), hepatic and skeletal muscle glycogen by 27.06% and 12.55% (doses of 800 mg), and increased plasma and pancreatic insulin content (31.77% and 52.34%, respectively) (Ighodaro and Akinloye, 2017). The limitation of Ong et al. study is that changes in insulin levels may be a confounding factor, and cynaroside leads to a slight increase in insulin levels, which in turn can increase GLUT 4 translocation and glycogen synthesis, while inhibiting gluconeogenesis enzymes such as G6Pase. Ighodaro et al. did not isolate and purify the bioactive compounds in the extract of Sapium ellipticum leaves, nor did they evaluate its long-term therapeutic effects and potential toxicity.

### 5.4 Diabetic complications

Due to the chronic elevation of blood glucose levels, long-term vascular complications of diabetes occur, including “microvascular diseases” (caused by damage to small vessels, such as retinopathy, nephropathy, and nerve damage) and “macrovascular diseases” (caused by damage to arteries, including acute cardiovascular and cerebrovascular diseases). The organ failure caused by diabetic complications is the most destructive consequence of diabetes and the primary cause of death in diabetic patients (Forbes and Cooper, 2013). It is reported that dyslipidemia is particularly important for the occurrence of neuropathy, retinopathy and nephropathy, lipid reduction can significantly improve diabetes complications and their prognosis (Davis et al., 2008). In T2DM, a large number of peripheral neuropathy cases (up to 10%–20% of patients) appear at the time of diagnosis (Charles et al., 2011). Increased lipid levels and body mass index may exacerbate the risk of diabetic neuropathy (Sone et al., 2005).

Aldose reductase (ALR) is the rate-limiting enzyme in the polyol pathway and its main role is to reduce glucose to whole sugar and participate in the regulation of the intracellular redox system (Gupta, 2024). Increased sorbitol production in many tissues does not readily diffuse through the cell membrane, and the intracellular accumulation of sorbitol has been associated with chronic complications of diabetes, such as cataracts, neuropathy and retinopathy (Sone et al., 2005). Glycoification is a non-enzymatic browning reaction caused by the amino-group reaction between reducing sugars and amino groups of proteins or lipids, which leads to the chemical modification of tissue proteins, known as advanced glycosylation end products (AGEs), leading to the dysfunction of proteins (Irani et al., 2014). Moreover, both diabetes and aging are associated with the accumulation of AGEs in tissues, increased oxidative stress and decreased antioxidant status. Complex fluorescent AGE molecules formed during the Maillard reaction can lead to protein cross-linking and promote the development and progression of diabetic complications, such as peripheral neuropathy, cataract, impaired wound healing, vascular injury, arterial wall sclerosis, and reduced myocardial compliance (Ott et al., 2014).

Plioukas et al. (2016) found that cynaroside inhibited the activity of ALR2. Since ALR is one of the pathogenic factors of diabetes, this study evaluated the inhibitory ability of cynaroside on ALR and verified its potential to combat long-term complications of diabetes. The antioxidant capacity of the above ground extract of *Tephrosia humilis* was assessed by DPPH radical and Co (II)/EDTA-induced chemiluminescence tests. The inhibitory activity of the extract on ALR2 and ALR1 was evaluated using partially purified mouse lens ALR2 and mouse kidney ALR1. Experimental results indicated that all extracts showed significant antioxidant capacity, and phytochemical analysis revealed that the active components in the extract contained various flavonoid compounds, including cynaroside (Plioukas et al., 2016). Hwang et al. (2019) assessed the inhibitory activity of ALR, late AGEs and the scavenging activity of DPPH radicals from 22 Peruvian plant extracts, and found cynaroside was the active ingredient to inhibited the activity of ALR. These two studies provide preliminary evidence of the antioxidant and aldose reductase inhibitory properties of cynaroside, but do not address the effects of cynaroside *in vivo* models, limiting a comprehensive assessment of its potential medicinal value in plants.

Diabetic vascular complications are early characterized by endothelial dysfunction, which is characterized by enhanced oxidative stress and inflammation, reduced bioavailability of NO and impaired endothelium-dependent vasodilatation. Acetylcholine-induced vasodilatation occurs through the Ach/JAK2/IRS-1/PI3K/Akt/eNOS pathway, and this pathway is disrupted in diabetic conditions (Huang et al., 2018). Moreover, in diabetic patients, microvascular injury occurred earlier than large vessel injury (Shi and Vanhoutte, 2017). Li et al. (2020a) suggested that cynaroside, the active component of *Coreopsis tinctoria* Nutt. flower, has a role of diabetic endothelial protection and may be related to its effect on the JAK2/IRS-1/PI3K/Akt/eNOS pathway, the associated oxidative stress and inflammatory response. Vascular function was assessed by examining endothelium-dependent vasodilatation and tail artery pressure in rat mesenteric arteries using a HFD and STZ-induced diabetes model. The results indicated that the treatment of cynaroside significantly improved endothelium-dependent vasodilation in the mesenteric arteries of diabetic rats, with a maximum relaxation of 79.82% in the control group and 91.87% in the extract-treated group (*p* < 0.01). The extract treatment also reduced tail artery pressure of rat, with both systolic and mean arterial pressure decreased (*p* < 0.05). A high glucose-induced human umbilical vein endothelial cells model was established *in vitro*, and the expression of IRS-1, Akt, and eNOS increased p-IRS-1Ser307, p-AktSer473, and p-eNOSSer1177 and decreased the expression of NOX4, TNF-α, IL-6, sVCAM, sICAM, and NF-κB (*p* < 0.01). Although Li et al. explored the mechanisms of action of active ingredients through *in vitro* experiments and animal models, their research lacks in-depth studies on the pharmacokinetics and pharmacodynamics of these components in the human body. The randomized controlled trials did not provide a detailed comparison of the effects of glucose at different concentrations and treatment durations on cells, which may result in an incomplete understanding of the mechanisms of endothelial cell damage induced by hyperglycemia.

## 6 Pharmacokinetics and toxicology of cynaroside

In the process of new drug development, pharmacokinetic studies have become an important part of drug preclinical and clinical researches. It not only plays an auxiliary role in drug toxicity or clinical studies, but also helps to optimize the screening of drug candidates, providing a new approach for studying modern drug therapy. Lin et al. (2015) isolated cynaroside from an ethanol extract of *Dendranthema morifolium* Ramat Tzvel and used it in animal studies with healthy adult male Sprague-Dawley rats. The pharmacokinetics of cynaroside in rats were studied by intravenous injection (10 mg/kg) and oral administration (1 g/kg). After intravenous injection of 10 mg/kg cynaroside, the area under curve (AUC) was 229 ± 15 min μg/mL. After oral administration of 1 g/kg, the AUC was 2,109 ± 350 min μg/mL, and the oral bioavailability was about 10% ± 2%. The biotransformation products of luteolin were detected in the oral cynaroside group, but not in the intravenous cynaroside group. The bioconversion ratio (metabolite/matrix compound AUC ratio) of luteolin to cynaroside was approximately 48.78% ± 0.12%. These results show that cynaroside is mainly hydrolyzed to luteolin in the intestinal mucosa, which is then absorbed by the systemic circulation.

Using an optimized ultra-high performance liquid chromatography-tandem mass spectrometry (UPLC-MS/MS) method, Wei et al. (2017) analyzed the pharmacokinetic parameters of cynaroside in normal and diabetic rats. Diabetic models were induced by STZ method, and rats were randomly divided into normal group and diabetic group, with six rats in each group. *Maydis stigma* extract was administered orally to rats at a dose of 5 g/kg, and blood samples were collected at different time points before and after administration. The results of normal group: area under the plasma concentration-time curve from time 0 to t (AUC_0-t_) 1,491 ± 341.1 μgh/l, area under the plasma concentration-time curve from time 0 to ∞ (AUC_0-_ ∞ ) 1,492 ± 341.2 μgh/l, the sum mean residence time 4.28 ± 0.15 h, peak plasma concentration (C_max_) 397.0 ± 78.27 μg/L, time to reach the maximum plasma concentration (T_max_) 0.83 ± 0.26 h, plasma clearance 17.22 ± 4.251 L/kg/h. The results of diabetic group: area under the plasma concentration-time curve from time 0 to t (AUC_0-t_) 3,072 ± 675.7 μgh/l, area under the plasma concentration-time curve from time 0 to ∞ (AUC_0-_ ∞ ) 3,074 ± 676.2 μgh/l, the sum mean residence time 4.53 ± 0.15 h, peak plasma concentration (C_max_) 769.3 ± 111.2 μg/L, time to reach the maximum plasma concentration (T_max_) 0.83 ± 0.26 h, plasma clearance 8.323 ± 1.966 L/kg/h. Compared to the normal group, the AUC_0-t_, AUC_0-_ ∞ , C_max_, and mean residence time of the T2DM group were significantly higher than those of the normal control group (*p* < 0.05). In the T2DM group, a longer T_max_ for the analyte was observed (*p* < 0.05), indicating slower absorption of the analyte. Results showed that cynaroside absorption increased and distribution and elimination processes slowed in the diabetic group compared to the normal group. These results may be attributed to pathological states of the gut, such as delayed gastric emptying and intestinal stagnation (Boland et al., 2013), small bowel hyperplasia and mucosal hypertrophy, which may lead to increased absorption of these analytes.

To investigate the effects of cynaroside on hepatocyte viability and proliferation, Zhu et al. cultured separately LO2 cells and primary liver cells with different concentrations (5–40 μM) of cynaroside for 12 or 24 h. The results showed that cynaroside significantly increased cell survival rates in both cell types (P < 0.05). Notably, the 20 μM treatment at 24 h demonstrated the most significant effect (P < 0.01), outperforming even the positive control drug at 20 μM. Additionally, cynaroside exhibited good safety profile, with the IC50 value for primary liver cells reaching 158.61 μM after 24 h treatment (Zhu et al., 2021). Gebhardt et al. found no significant toxicity of artichoke leaf extract below 0.2 mg/mL to HepG2 cells and rat primary hepatocytes (viability > 90%), and only showed significant toxicity at higher concentrations (>5 mg/mL) by MTT assay (Gebhardt, 1998; 2002). Tabrez et al. (2021) used the THP-1 differentiated macrophages line to assess the cytotoxicity of cynaroside. The cell viability was evaluated by MTT assay, and the 50% cytotoxicity concentration (CC_50_) of allium glycosides was 65.33 ± 5.272 μM, while that of mitifosine was 20.39 ± 1.69 μM. Yang et al. conducted a 12-week intervention in mice by orally administering cynaroside at different doses (25, 50, and 100 mg/kg). The study demonstrated that cynaroside exhibited no significant toxic side effects while improving glucose-lipid metabolism disorders in obese mice, reducing inflammatory responses, enhancing gut microbiota diversity, and stabilizing the intestinal microbial balance (Yang et al., 2025). Tabrez et al. also used Lipinski’s rules, Molsoft L.L.C., and Swiss ADME database to evaluate the drug properties of cynaroside, including absorption, distribution, metabolism, excretion, and toxicity. Cynaroside follows Lipinski’s rules and has good oral bioavailability. It is predicted to be an enzyme inhibitor having sufficient solubility and non-toxic properties (Tabrez et al., 2021).

In general, cynaroside showed good safety (no obvious toxicity) and high oral bioavailability. It was mainly hydrolyzed by intestinal mucosa to its metabolite luteolin, which was then absorbed into the systemic circulation. These characteristics laid a good foundation for its clinical application.

## 7 Clinical research progress of cynaroside

Cynaroside, as a glycosylation product of luteolin, is present in a variety of foods, vegetables and medicinal herbs, and is commonly used in dietary supplements due to its health benefits. Meanwhile, cynaroside is an indicator component for the quality control of honeysuckle in the pharmacopoeia (Liu et al., 2024). Cynaroside exhibits a wide range of pharmacological effects, including treatment of abnormalities in fat metabolism, anti-inflammatory, antioxidation, hepatoprotective, and antidiabetic activities (Lu et al., 2024). At present, patients clinically consume cynaroside through cynaroside-rich traditional Chinese medicine compound preparations or healthcare products with identical medicine and food. Clinical trials using cynaroside are yet to be further investigated.

Jia et al. (2022) found the relieving effect of Mailuoning Oral Liquid solution on MASLD. Mailuoning Oral Liquid is a modern traditional Chinese medicine prescription composed by *Lonicerae japonicae* flos, Radix *Achyranthis Bidentatae*, Radix *Scrophulariae* and *Dendrobium* Caulis. Mailuoning Oral Liquid is generally used to treat the syndrome of blood stasis in clinical practice. Research has confirmed that Mailuoning Oral Liquid can alleviate MASH by reducing lipid accumulation in the liver, inhibiting inflammation and alleviating fibrosis through activating the PGC-1α/PPARα signaling pathway in the MCD diet-induced mouse model (Jia et al., 2022). In particular, two omponents of traditional Chinese medicine, *L*. *japonicae* flos (honeysuckle) and Radix *Achyranthis Bidentatae* (buckwheat), also significantly contribute to the effectiveness of Mailuoning Oral Liquid in suppressing MASLD (Jia et al., 2022).

*L*. *japonicae* Flos, also called Jinyinhua in China, comes from the dried flower buds or flowers to be opened of *L*. *japonicae* in the *Lonicera* family. It has a long history of medicinal use and possesses a wide range of application prospects (Li et al., 2020b). *L*. *japonicae* flos is a renowned Chinese herbal medicine, first documented in Shen Nong’s Herbal Classic (Shennong Bencao Jing) and classified as a top-quality herb (Ma et al., 2024). Yokota (2017) have developed preventive drugs from food and natural ingredients to treat scientifically based diseases. They have found that *L*. *japonicae* flos has good results in treating metabolic syndrome and liver disease, and have tested this idea in animal models of disease. Based on these findings, Yokota et al. planned further studies, including clinical studies in human participants (Yokota, 2017). Kemertelidze et al. conducted chemical studies on plant *Satureja hortensis* L., which is rich in phenolic compounds such as cynaroside. Pharmacological investigation of the extract of *S*. *hortensis* was carried out on intact animals and animals with experimental alloxan diabetes, and antidiabetic Arfasetine was chosen as a reference drug. The study revealed that the extract of *S*. *hortensis* exhibited hypoglycemic activity and significantly reduced blood sugar levels, and was safe in the long run (Kemertelidze et al., 2012). Drug dosage form named saturin-capsules containing 0.33 g of dried aqueous extract of *S*. *hortensis* leaves has been developed. Clinical trials of saturin confirmed its effiacy in T2DM (Kemertelidze et al., 2012). The drug is registered by the Ministry of Health, Labour and Social Welfare of Georgia (registration certificate No.003658) and approved for use in T2DM either independently or in combination with other hypoglycemic agents (Kemertelidze et al., 2012).

Overall, cynaroside, a key active ingredient in traditional Chinese medicines like honeysuckle, has been used in clinical practice for a long time through compound formulas (e.g., Sanren Tang, Mailuoning oral liquid). However, research on its monomer form is still in its early stages, with current findings primarily focusing on animal models and cell experiments. Recognizing the importance of clinical trial registration, we specifically reviewed the Chinese Clinical Trial Registration Center (https:www.chictr.org.cn/showproj.html?proj=219472) and the International Clinical Trials Registry Platform (https://trialsearch.who.int/). However, no clinical studies were found by using the keyword “cynaroside, luteolin-7-O-glucoside, luteoloside, cinaroside” for precise searches.

## 8 Challenges and future directions for cynaroside in clinical applications

Cynaroside is a flavonoid widely found in plants. It has significant economic advantages over other natural ingredients, and its content in plants is high (Wang et al., 2017a), which has more industrialization potential than many trace active ingredients. Its extraction methods are very mature. Its biological activity has been studied comprehensively and can therefore be easily applied in clinical studies (Bouyahya et al., 2023). The methods of separation and purification are relatively few, including macroporous resin column chromatography purification, molecular imprinting technology purification and polyamide column chromatographic separation (Li et al., 2020b). The use of macroporous resin for the separation and purification of cynaroside is simple and low cost, and is now widely used. However, there are problems such as excessive time consumption, impurity contamination of macropore adsorptive resin D101 and other resins, and decreased adsorption efficiency after repeated use (Wang et al., 2017b; Chen et al., 2024). Thus, it is necessary to continuously optimize or update the separation technology to make it more simple and efficient to improve the production of cynaroside, so as to promote the better service of cynaroside for clinical treatment in the future.

In addition, cynaroside and other natural compounds must overcome challenges, such as bioavailability, safety and lack of clinical evidence, before they can be successfully incorporated into medical practice. Importantly, the protective properties of cynaroside for lowering blood sugar and lipids and for multiple organs cannot be achieved entirely by dietary supplementation due to its inability to achieve effective blood concentrations. Currently, pharmaceutical preparations purified by cynaroside are commercially available only as analytical standards (Caporali et al., 2022). To address these problems, advanced delivery systems such as nanoparticles and liposomes offer promising solutions by improving the bioavailability and stability (Lin et al., 2022).

Meanwhile, the metabolism process and the mechanism of efficacy in the body of cynaroside are not completely clear. The occurrence of lipometabolic diseases involves many key genes and pathways, such as PPARγ pathway, CYP7A1 gene, etc. The relationship between these key molecules, signaling pathways and cynaroside in lipid metabolic diseases has only been initially investigated. It has been shown that combining cynaroside with other natural compounds could potentially enhance the cardioprotective effects during Doxorubicin chemotherapy (Yao et al., 2016). And the combination with cynaroside can also enhance the efficacy of radiotherapy or chemotherapeutic drugs in neoplastic diseases, suggesting the advantages and possibilities of combined treatment strategies (Zou et al., 2024). Similarly, cynaroside may be further investigated in conjunction with other drugs or treatments for lipid-related diseases. Furthermore, conducting comprehensive clinical trials is essential to evaluate the efficacy and safety of these natural compounds. To date, there is a significant lack of clinical translation studies for the monomer cynaroside, without related clinical trial registered, and no comprehensive reports on human efficacy and safety have been published.

## 9 Summary

This article reviews the pharmacological effects of cynaroside, a natural flavonoid, on lipid metabolism and related diseases. It presents data from molecular, cellular, and animal studies. The review claims to offer a comprehensive mechanistic summary and outlines the compound’s potential clinical applications. Although its extraction method has been mature, the study of its monomer is still in the initial stage, and there is no complete human efficacy and safety evaluation report, which poses a major challenge to the clinical promotion of cynaroside. Therefore, further elucidating the molecular mechanisms of cynaroside, reducing costs and promoting its clinical application are key directions for future research. Additionally, the potential synergistic or antagonistic interactions between cynaroside and other drugs or treatments warrant further investigation.
